# Thiamine metabolism is critical for regulating correlated growth of dendrite arbors and neuronal somata

**DOI:** 10.1038/s41598-017-05476-w

**Published:** 2017-07-13

**Authors:** Huimin Liu, Shaoming Sang, Yuan Lu, Zhongfeng Wang, Xiang Yu, Chunjiu Zhong

**Affiliations:** 1 Institutes of Brain Science & Collaborative Innovation Center for Brain Science; Department of Neurology, Zhongshan Hospital; State Key Laboratory of Medical Neurobiology, Fudan University, Shanghai, 200032 China; 2 School of Life Sciences, Fudan University, Shanghai, 200438 China; 3 Institute of Neuroscience, State Key Laboratory of Neuroscience, CAS Center for Excellence in Brain Science and Intelligence Technology, Chinese Academy of Sciences, Shanghai, 200031 China

## Abstract

Thiamine is critical for cellular function, as its phosphorylated and active form, thiamine diphosphate (TDP), acts as coenzyme for three key enzymes in glucose metabolism. Mutations in thiamine transporter, TDP synthesizing enzyme or carrier, including solute carrier family 19 member 3 (*SLC19A3*), thiamine pyrophosphokinase (*TPK1*) and solute carrier family 25 member 19 (*SLC25A19*), have been associated with developmental neurological disorders, including microcephaly and Leigh syndrome. However, little is known about how thiamine metabolism regulates neuronal morphology at the cellular level. Here, using primary rat hippocampal neuronal cultures, we showed that reducing the expression of *Tpk1*, *Slc25a19* or *Slc19a3* in individual neurons significantly reduced dendrite complexity, as measured by total dendritic branch tip number (TDBTN) and total dendritic branch length (TDBL). The specificity of the RNAi effects were verified by overexpression of RNAi resistant human constructs. Importantly, changes in both TDBTN and TDBL tightly correlated with reduction in soma size, demonstrating coordinated regulation of soma and dendrite growth by thiamine. The requirement of thiamine metabolism for coordinated somata and dendrite growth is highly consistent with the microcephaly and neurodegenerative phenotypes observed in thiamine loss-of-function diseases.

## Introduction

Thiamine (T), also known as thiamin or Vitamin B1, is a water-soluble B vitamin that upon uptake, is rapidly phosphorylated by thiamine pyrophosphokinase (TPK1) in the cytoplasm to form thiamine diphosphate (TDP)^1–3^. TDP plays a vital role in glucose metabolism as the coenzyme for three key enzymes: pyruvate dehydrogenase complex (PDHC) and α-ketoglutarate dehydrogenase complex (KGDHC) in the Krebs cycle and transketolase (TK) in the pentose phosphate pathway^4, 5^. Other molecules important to its metabolism and function include solute carrier family 19 member 3 (SLC19A3), a high-affinity transporter for thiamine in the cell membrane^6^, and solute carrier family 25 member 19 (SLC25A19), a six-transmembrane protein that serves as the mitochondrial transporter of TDP^7–9^.

Thiamine is critical to the individual’s health, as its extended deficiency in food sources can result in beriberi and Wernicke-Korsakoff syndrome, with severe manifestations in the peripheral nervous system and brain respectively^3, 10, 11^. Furthermore, mutations in thiamine metabolism genes *SLC19A3*, *SLC25A19* and *TPK1* ^5, 7, 12, 13^ have been reported to result in Leigh syndrome, a progressive neurodegenerative disorder of early childhood. Moreover, a point mutation in a conserved residue in *SLC25A19* that affects its transporter function, has been identified in congenital microcephaly, a severe form of encephalopathy with brain malformations^14^. Consistently, *Slc25a19* knockout mice were embryonic lethal and had CNS malformations^15^.

The above-described genetic data strongly point to important roles of thiamine metabolism in CNS development. However, little is known about the function of this pathway at the cellular level. Here, we approach this question by knocking down each of three molecules key to thiamine metabolism, namely *Tpk1*, *Slc25a19* and *Slc19a3*, and assaying the resulting effect on neuronal morphology using dissociated primary hippocampal neuronal cultures. Our results showed that all three genes had important functions in coordinately regulating dendritic arborization and neuronal soma size. These effects were mimicked by pharmacological inhibition of thiamine metabolism, and could be rescued by overexpression of RNAi-resistant human sequences, demonstrating specificity of the phenotypes and conserved functions of the thiamine metabolizing enzymes. These results provide further evidence for a critical role of thiamine metabolism during neuronal development.

## Results

### *Tpk1* knockdown in hippocampal pyramidal neurons significantly reduces dendrite and soma growth

**Figure 1:**
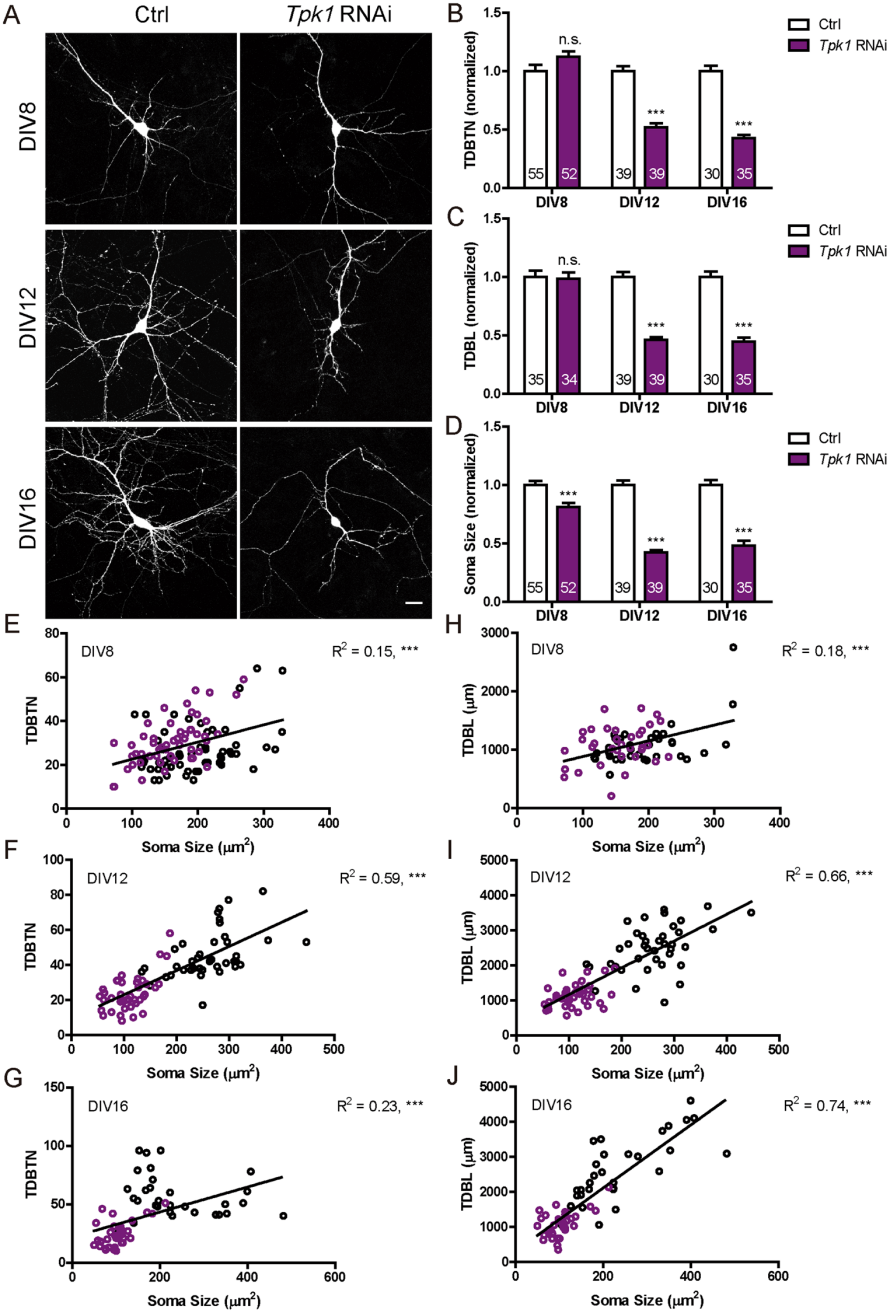
*Tpk1* knockdown in primary hippocampal neurons significantly reduced TDBTN, TDBL and soma size. (**A**) Representative images of primary hippocampal neurons transfected with control and *Tpk1* RNAi constructs, fixed at DIV8, DIV12 or DIV16. Scale bar: 20 μm. (**B**) Quantitation of TDBTN: *Tpk1* RNAi at DIV8 (1.12 ± 0.05, n.s.), DIV12 (0.52 ± 0.04, *P* < 0.001), DIV16 (0.43 ± 0.03, *P* < 0.001). (**C**) Quantitation of TDBL: *Tpk1* RNAi at DIV8 (0.99 ± 0.05, n.s.), DIV12 (0.46 ± 0.02, *P* < 0.001), DIV16 (0.45 ± 0.03, *P* < 0.001). (**D**) Quantitation of soma size: *Tpk1* RNAi at DIV8 (0.81 ± 0.03, *P* < 0.001), DIV12 (0.42 ± 0.02, *P* < 0.001), DIV16 (0.48 ± 0.04, *P* < 0.001). (**B**–**D**) Two way ANOVA followed by Bonferroni’s post-test. (**E**–**G**) Significant correlations exist between TDBTN and soma size at DIV8 (**E**, *n* = 107, R^2^ = 0.15, *P* < 0. 001), DIV12 (**F**, *n* = 78, R^2^ = 0.59, *P* < 0. 001) and DIV16 (**G**, *n* = 65, R^2^ = 0.23, p < 0. 001). (**H**–**J**) Significant correlations are observed between TDBL and soma size at DIV8 (**H**, *n* = 69, R^2^ = 0.18, *P* < 0.001), DIV12 (**I**, *n* = 78, R^2^ = 0.66, *P* < 0.001) and DIV16 (**J**, *n* = 65, R^2^ = 0.74, *P* < 0.001). In (**E**–**J**) Black circles represent control neurons, while purple circles represent *Tpk1* RNAi neurons. In this and all subsequent figures, “*n*” represents the number of neurons and is as indicated inside bar graphs, error bars represent s.e.m.; *P < 0.05, **P < 0.01, ***P < 0.001, n.s. *P* > 0.05.

We first investigated the function of Tpk1, the kinase that catalyzes the transfer of two phosphate groups from adenosine triphosphate (ATP) to thiamine to produce TDP^16, 17^, the active form of thiamine. Having shown that the RNAi sequence effectively reduced Tpk1 protein level (Fig. S1A), as well as the intracellular level of TDP (Fig. S1B), we assayed its role in regulating neuronal morphology by transfecting DIV 6 high density primary rat hippocampal neurons cultures sparsely with *Tpk1* RNAi using the calcium phosphate method, together with GFP as a marker of neuronal morphology. Neurons were fixed and assayed at 2, 6 or 10 days following transfection. As compared to neurons transfected with the control RNAi construct, those expressing *Tpk1* RNAi had significantly lower dendrite complexity on DIV 12 and DIV 16, but not at the earlier time point of DIV 8 (Fig. 1A–C), as measured by reduced total dendritic branch tip number (TDBTN) (Fig. 1B) and total dendritic branch length (TDBL) (Fig. 1C). In terms of absolute numbers, TDBTN for control neurons increased from 27.15 ± 1.53 at DIV 8 to 45.85 ± 2.19 at DIV 12 and 57.03 ± 3.36 at DIV 16, doubling in complexity, while TDBTN for *Tpk1* RNAi neurons essentially remained at the same level during this period (30.67 ± 1.50 for DIV 8, 23.82 ± 1.65 for DIV 12 and 24.66 ± 1.85 for DIV 16). Similarly, TDBL for control neurons increased from 1095.41 ± 61.64 μm at DIV 8 to 2495.64 ± 106.68 μm at DIV 12 and 2623.22 ± 168.87 μm at DIV 16, while TDBL for *Tpk1* RNAi neurons remained at the same level (1079.10 ± 58.40 μm for DIV 8, 1139.49 ± 53.10 μm for DIV 12 and 1088.77 ± 61.21 μm for DIV 16). Together, these results suggest a critical function of Tpk1 in promoting dendrite growth and arborization.

During the analysis of dendrite arborization, we noticed that *Tpk1* RNAi neurons had significantly smaller soma. We thus also quantified this parameter, and found its changes to be very dramatic, with significant reduction already at DIV 8 and increasing in proportion at DIV 12 and 16 (Fig. 1A,D). In terms of absolute numbers, soma size for control neurons were 199.32 ± 7.77 μm^2^ at DIV 8, and increased to 261.11 ± 10.10 μm^2^ at DIV 12 and 236.48 ± 18.02 μm^2^ at DIV 16, while those for *Tpk1* RNAi actually reduced from 160.16 ± 6.43 μm^2^ at DIV 8 to 110.36 ± 5.57 μm^2^ at DIV 12 and 102.90 ± 5.88 μm^2^ at DIV 16. In other words, soma size reduction in *Tpk1* RNAi neurons occurred as early as DIV 8 and continued to become smaller as the neurons aged.

Since soma size changed in the same direction as dendrite complexity and started from an even earlier time point, we examined if these changes were related. The results showed that for both control and *Tpk1* RNAi neurons, TDBTN and TDBL were highly correlated with soma size, at all time points examined (Fig. 1E–J), suggesting that *Tpk1* was critical for regulating both parameters in a coordinated fashion.

### Overexpression of *TPK1* significantly rescues the reduction of dendritic complexity and soma size induced by *Tpk1* RNAi

**Figure 2:**
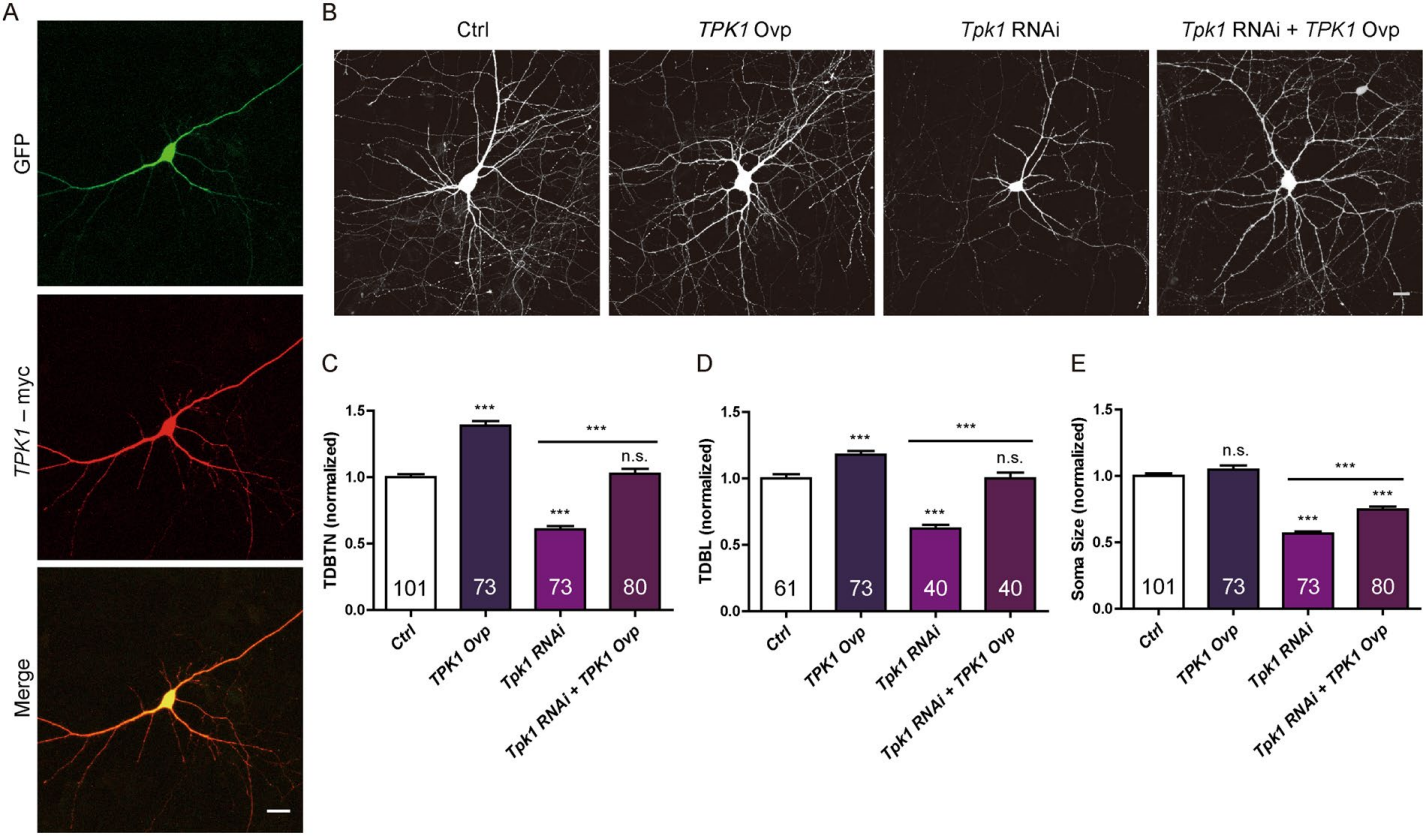
Overexpression of human *TPK1* significantly rescued the reduction of TDBTN, TDBL and soma size induced by *Tpk1* RNAi. (**A**) Immuostaining of DIV8 neuron transfected with GFP and *TPK1*-Myc constructs. (**B**) Representative images of DIV10 neurons, conditions as indicated. (**C**) Quantitation of TDBTN: Ctrl (1.00 ± 0.02), *TPK1* Ovp (1.39 ± 0.04, *P* < 0.001 vs Ctrl), *Tpk1* RNAi (0.61 ± 0.02, *P* < 0.001 vs Ctrl), *Tpk1* RNAi + *TPK1* Ovp (1.03 ± 0.04, n.s. vs Ctrl, *P* < 0.001 vs *Tpk1* RNAi). (**D**) Quantitation of TDBL: Ctrl (1.00 ± 0.03), *TPK1* Ovp (1.18 ± 0.03, *P* < 0.001 vs Ctrl), *Tpk1* RNAi (0.62 ± 0.03, *P* < 0.001 vs Ctrl), *Tpk1* RNAi + *TPK1* Ovp (1.00 ± 0.05 n.s. vs Ctrl, *P* < 0.001 vs *Tpk1* RNAi). (**E**) Quantitation of soma size: Ctrl (1.00 ± 0.02), *TPK1* Ovp (1.05 ± 0.03, n.s. vs Ctrl), *Tpk1* RNAi (0.56 ± 0.02, *P* < 0.001 vs Ctrl), *Tpk1* RNAi + *TPK1* Ovp (0.75 ± 0.02, *P* < 0.001 vs Ctrl, *P* < 0.001 vs *Tpk1* RNAi). One-way ANOVA followed by Tukey’s post-test. Scale bar: 20 μm.

To determine if regulation of dendrite arborization and soma size by *Tpk1* was bidirectional, we overexpressed human *TPK1* (Fig. 2A) and also used it to rescue the effects of *Tpk1* RNAi. Neurons were transfected at DIV6 and examined at DIV 10. Overexpression of *TPK1* significantly increased TDBTN and TDBL, demonstrating that *Tpk1* bidirectionally regulated dendrite development (Fig. 2B–D). *TPK1* overexpression (*Tpk1* RNAi resistant) in *Tpk1* RNAi neurons also significantly rescued reductions in TDBTN, TDBL and soma size (Fig. 2B–E), demonstrating specificity of the RNAi effects.

### *Slc25a19* knockdown significantly reduces dendritic and soma growth

**Figure 3:**
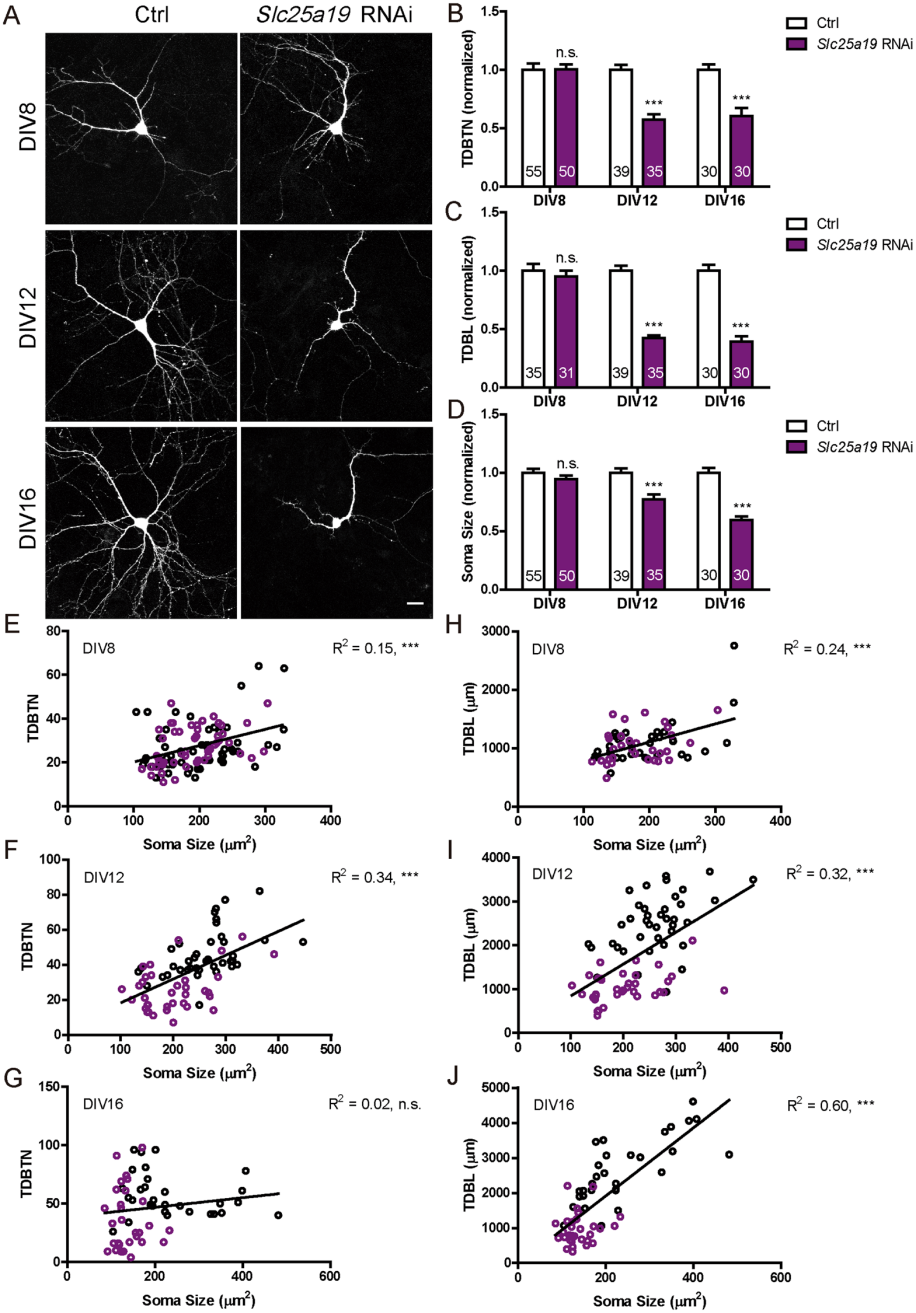
*Slc25a19* knockdown in primary hippocampal neurons significantly reduced TDBTN, TDBL and soma size. (**A**) Representative images of primary hippocampal neurons transfected with control and *Slc25a19* RNAi plasmids, fixed at DIV8, DIV12 or DIV16. Scale bar: 20 μm. (**B**) Quantitation of TDBTN: *Slc25a19* RNAi at DIV8 (1.00 ± 0.04, n.s.), DIV12 (0.57 ± 0.05, *P* < 0. 001), DIV16 (0.61 ± 0.07, *P* < 0. 001). (**C**) Quantitation of TDBL: *Slc25a19* RNAi at DIV8 (0.95 ± 0.05, n.s.), DIV12 (0.42 ± 0.02, *P* < 0. 001), DIV16 (0.39 ± 0.05, *P* < 0. 001). (**D**) Quantitation of soma size: *Slc25a19* RNAi at DIV8 (0.95 ± 0.03, n.s.), DIV12 (0.77 ± 0.04, *P* < 0. 001), DIV16 (0.59 ± 0.03, *P* < 0. 001). (**B**–**D**) Two way ANOVA followed by Bonferroni’s post-test. Data for control neurons same as that shown in Fig. 1. (**E**–**G**) Significant correlations exist between TDBTN and soma size at DIV8 (**E**, *n* = 105, R^2^ = 0.15, *P* < 0. 001), DIV12 (**F**, *n* = 74, R^2^ = 0.34, *P* < 0. 001) but not DIV16 (**G**, *n* = 60, R^2^ = 0.02, n.s.). (**H**–**J**) Significant correlations are observed between TDBL and soma size at DIV8 (**H**, *n* = 66, R^2^ = 0.24, *P* < 0. 001), DIV12 (**I**, *n* = 74, R^2^ = 0.32, *P* < 0. 001) and DIV16 (**J**, *n* = 60, R^2^ = 0.60, *P* < 0. 001). In (**E**–**J**) Black circles represent control neurons, while purple circles represent *Slc25a19* RNAi neurons.

We next examined the function of Slc25a19, the protein responsible for transporting TDP^7–9^ into the mitochondria, where TDP functions as a coenzyme for PDHC and KGDHC in the Krebs cycle. Neurons transfected with *Slc25a19* RNAi (verification in Fig. S1C) on DIV 6 had significantly reduced TDBTN, TDBL and soma size on DIV 12 and DIV 16 but not the earlier time point of DIV 8 (Fig. 3A–D). Furthermore, changes in TDBTN and TDBL were highly correlated with those of soma size for all comparisons, except for TDBTN at DIV 16 (Fig. 3E–J). These results were similar to those of *Tpk1* RNAi (Fig. 1), consistent with the two proteins being part of the same metabolic pathway.

### *SLC25A19* overexpression significantly rescues the reduction of dendritic complexity induced by *Slc25a19* RNAi

**Figure 4:**
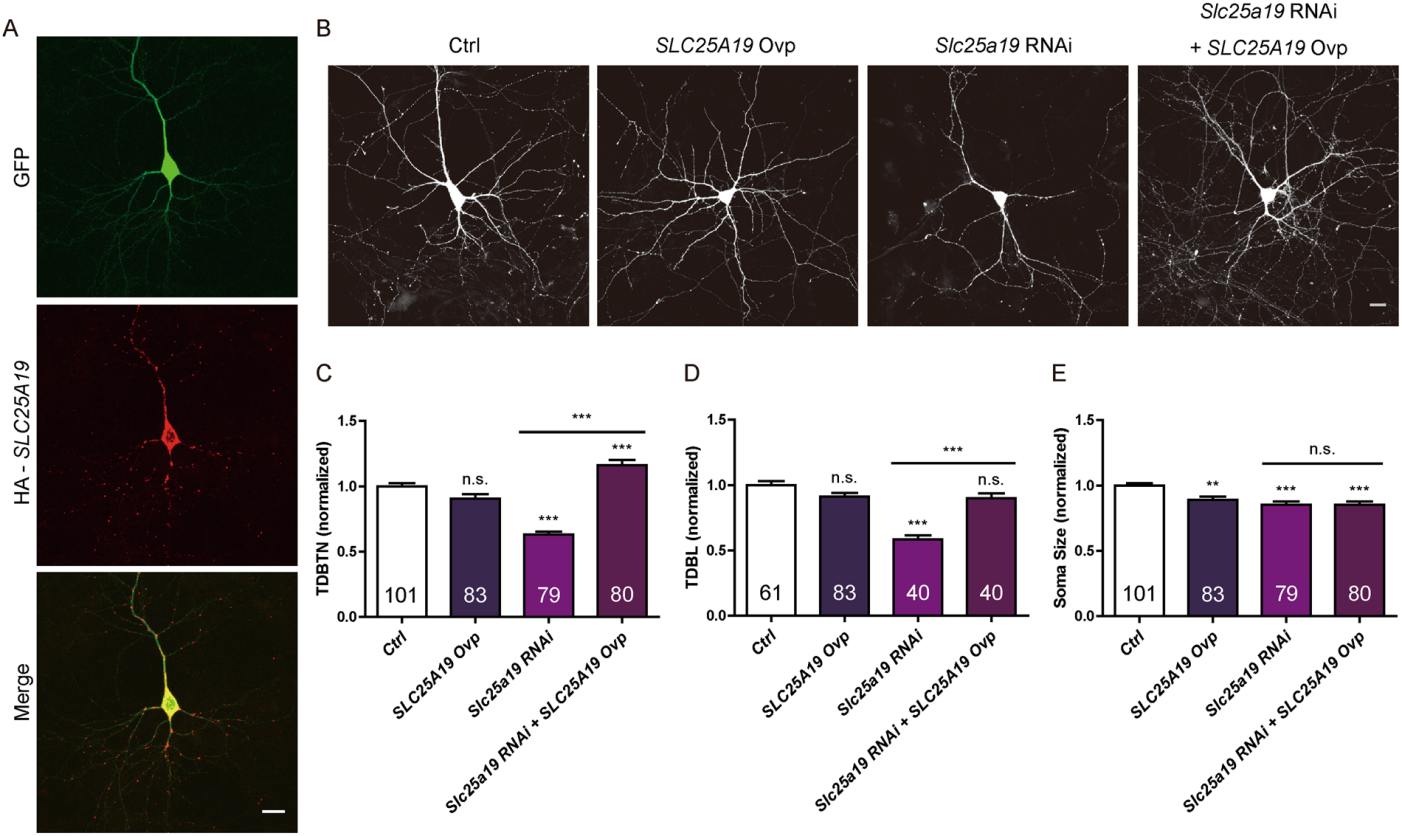
Overexpression of human *SLC25A19* significantly rescued the reduction of TDBTN and TDBL induced by *Slc25a19* RNAi. (**A**) Immuostaining of DIV11 neuron transfected with GFP and HA-*SLC25A19* constructs. (**B**) Representative images of DIV10 neurons, conditions as indicated. (**C**) Quantitation of TDBTN: Ctrl (1.00 ± 0.02), *SLC25A19* Ovp (0.91 ± 0.03, n.s. vs Ctrl), *Slc25a19* RNAi (0.63 ± 0.02, *P* < 0.001 vs Ctrl), *Slc25a19* RNAi + *SLC25A19* Ovp (1.16 ± 0.04, *P* < 0.001 vs Ctrl, *P* < 0.001 vs *Slc25a19* RNAi). (**D**) Quantitation of TDBL: Ctrl (1.00 ± 0.03), *SLC25A19* Ovp (0.91 ± 0.03, n.s. vs Ctrl), *Slc25a19* RNAi (0.58 ± 0.03, *P* < 0.001 vs Ctrl), *Slc25a19 RNAi* + *SLC25A19* Ovp (0.90 ± 0.04, n.s. vs Ctrl, *P* < 0.001 vs *Slc25a19* RNAi). (**E**) Quantitation of soma size: Ctrl (1.00 ± 0.02), *SLC25A19* Ovp (0.89 ± 0.03, *P* < 0.01 vs Ctrl), *Slc25a19* RNAi (0.85 ± 0.03, *P* < 0.001 vs Ctrl), *Slc25a19* RNAi + *SLC25A19* Ovp (0.85 ± 0.02, *P* < 0.001 vs Ctrl, n.s. vs *Slc25a19* RNAi). Data for control neurons same as that shown in Fig. 2. One-way ANOVA followed by Tukey’s post-test. Scale bar: 20 μm.

Overexpression of human *SLC25A19* (expression verification in Fig. 4A) alone did not significantly affect TDBTN or TDBL, but it was effective in rescuing the reduction in TDBL and TDBTN induced by *Slc25a19* RNAi (Fig. 4B–D). These results confirm the specificity of the *Slc25a19* RNAi. In terms of soma size, *SLC25A19* overexpression surprisingly induced a small but significant reduction as compared to control neurons, and did not rescue the *Slc25a19* RNAi condition (Fig. 4B,E). The relatively weak effect of *SLC25A19* overexpression may be related to its mitochondrial location, which provides TDP for enzymes PDHC and KGDHC, but does not affect the availability of TDP to cytoplasmic enzyme TK.

### *Slc19a3* knockdown significantly inhibits dendrite and soma growth

**Figure 5:**
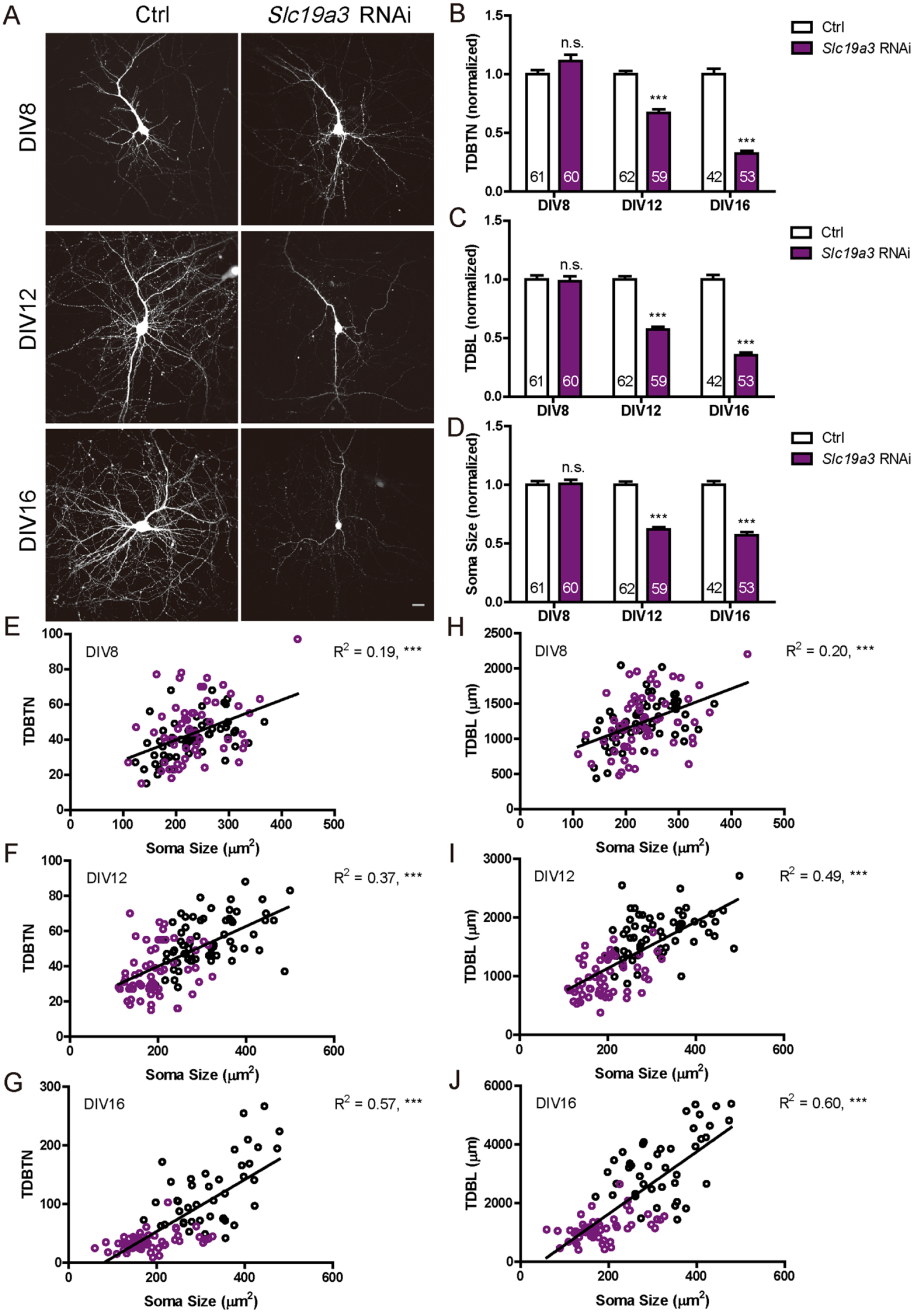
*Slc19a3* knockdown in primary hippocampal neurons significantly reduced TDBTN, TDBL and soma size. (**A**) Representative images of primary hippocampal neurons transfected with control and *Slc19a3* RNAi constructs, fixed at DIV8, DIV12 or DIV16. Scale bar: 20 μm. (**B**) Quantitation of TDBTN: *Slc19a3* RNAi at DIV8 (1.11 ± 0.05, n.s.), DIV12 (0.67 ± 0.03, *P* < 0. 001), DIV16 (0.32 ± 0.02, *P* < 0. 001). (**C**) Quantitation of TDBL: *Slc19a3* RNAi at DIV8 (0.98 ± 0.04, n.s.), DIV12 (0.57 ± 0.02, *P* < 0. 001), DIV16 (0.35 ± 0.02, *P* < 0. 001). (**D**) Quantitation of soma size: *Slc19a3* RNAi at DIV8 (1.01 ± 0.03, n.s.), DIV12 (0.62 ± 0.02, *P* < 0. 001), DIV16 (0.57 ± 0.03, *P* < 0. 001). (**B**–**D**) Two way ANOVA followed by Bonferroni’s post-test. (**E**–**G**) Significant correlations exist between TDBTN and soma size at DIV8 (**E**, *n* = 121, R^2^ = 0.19, *P* < 0. 001), DIV12 (**F**, *n* = 121, R^2^ = 0.37, *P* < 0. 001) and DIV16 (**G**, *n* = 95, R^2^ = 0.57, *P* < 0. 001). (**H**–**J**) Significant correlations are observed between TDBL and soma size at DIV8 (**H**, *n* = 121, R^2^ = 0.20, *P* < 0. 001), DIV12 (**I**, *n* = 121, R^2^ = 0.49, *P* < 0. 001) and DIV16 (**J**, *n* = 95, R^2^ = 0.60, *P* < 0. 001). In (**E**–**J**) Black circles represent control neurons, while purple circles represent *Slc19a3* RNAi neurons.

We next investigated the function of Slc19a3, a protein containing 12 putative transmembrane domains that serves as the high-affinity transporter for thiamine in the cell membrane^6^. *Slc19a3* RNAi expression (efficiency verified in Fig. S1D) from DIV 6 reduced TDBL, TDBTN and soma size at DIV 12 and 16, but not the earlier time point of DIV 8 (Fig. 5A–D). Furthermore, at all time points, changes in TDBL and TDBTN were correlated with those in soma size (Fig. 5E–J). These results are similar to those of *Tpk1* RNAi and *Slc25a19* RNAi, consistent with critical roles of all three proteins in thiamine metabolism.

### *SLC19A3* overexpression significantly rescues the reduction of dendritic complexity induced by *Slc19a3* RNAi

**Figure 6:**
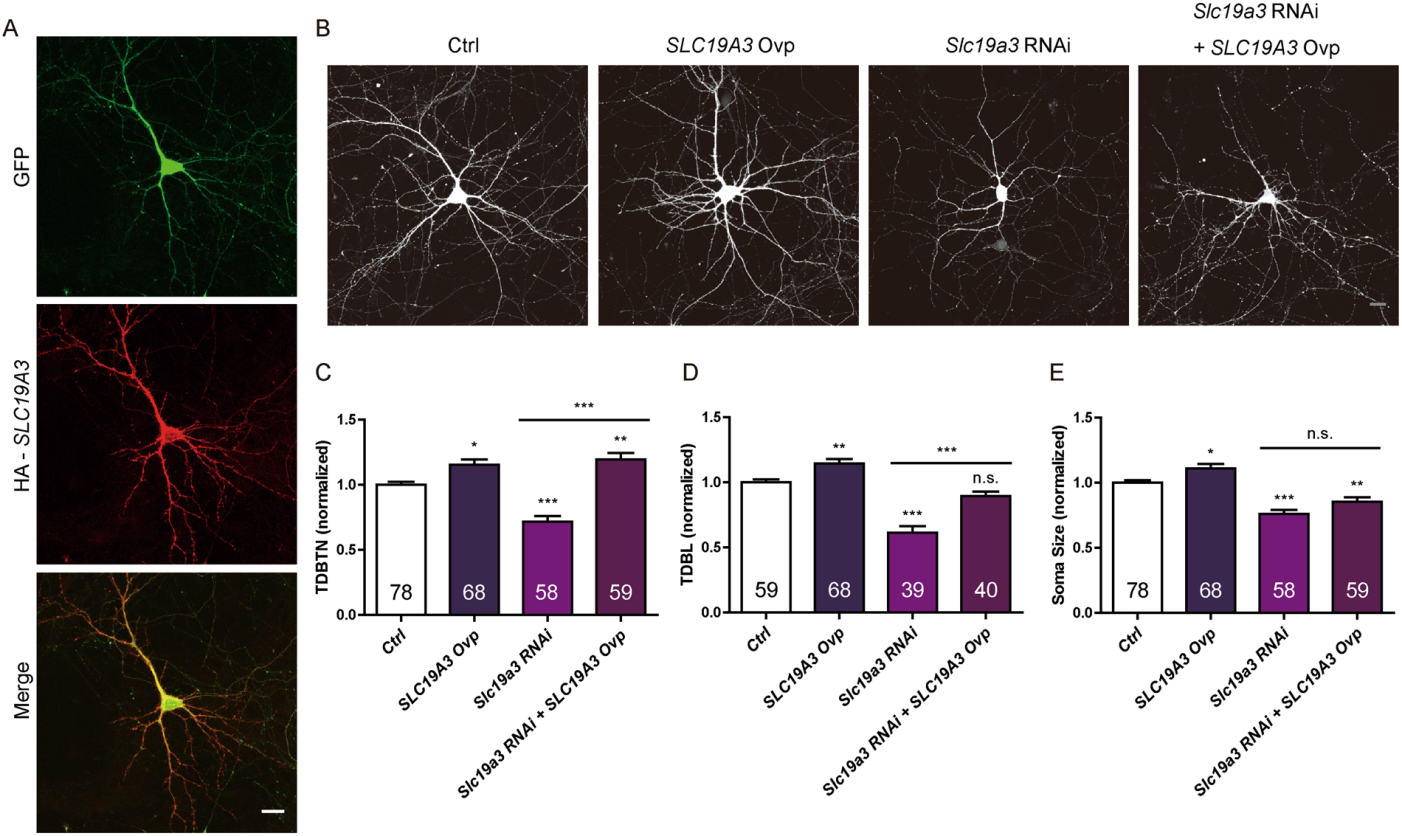
Overexpression of human *SLC19A3* significantly rescued the reduction of TDBTN and TDBL induced by *Slc19a3* RNAi. (**A**) Immuostaining of DIV11 neuron transfected with GFP and HA-*SLC19A3* constructs. (**B**) Representative images of DIV10 neurons, conditions as indicated. (**C**) Quantitation of TDBTN: Ctrl (1.00 ± 0.02), *SLC19A3* Ovp (1.16 ± 0.04, *P* < 0.05 vs Ctrl), *Slc19a3* RNAi (0.72 ± 0.04, *P* < 0.001 vs Ctrl), *Slc19a3* RNAi + *SLC19A3* Ovp (1.20 ± 0.05, *P* < 0.01 vs Ctrl, *P* < 0.001 vs *Slc19a3* RNAi). (**D**) Quantitation of TDBL: Ctrl (1.00 ± 0.02), *SLC19A3* Ovp (1.15 ± 0.03, *P* < 0.01 vs Ctrl), *Slc19a3* RNAi (0.61 ± 0.05, *P* < 0.001 vs Ctrl), *Slc19a3* RNAi + *SLC19A3* Ovp (0.90 ± 0.03, n.s. vs Ctrl, *P* < 0.001 vs *Slc19a3* RNAi). (**E**) Quantitation of soma size: Ctrl (1.00 ± 0.02), *SLC19A3* Ovp (1.11 ± 0.03, *P* < 0.05 vs Ctrl), *Slc19a3* RNAi (0.76 ± 0.03, *P* < 0.001 vs Ctrl), *Slc19a3* RNAi + *SLC19A3* Ovp (0.86 ± 0.03, *P* < 0.01 vs Ctrl, n.s. vs *Slc19a3* RNAi). One-way ANOVA followed by Tukey’s post-test. Scale bar: 20 μm.

Overexpression of human *SLC19A3* (expression verification in Fig. 6A) resulted in a small but significant increase in TDBTN, TDBL and soma size (Fig. 6B–E), and significantly rescued the effect of *Slc19a3* RNAi on TDBL and TDBTN, but not soma size (Fig. 6B–E). These results demonstrate specificity of the *Slc19a3* RNAi and the ability of Slc19a3 to bidirectionally regulate dendrite development.

### Non-redundancy between the function of pathway components

Having shown that Tpk1, Slc25a19 and Slc19a3 were all required for regulating dendrite development and soma size, we next asked if these molecules are non-redundantly required. In other words, could loss-of-function of one pathway component be rescued by overexpression of another component. Our results showed that overexpression of human *SLC25A19* or *SLC19A3* in *Tpk1* RNAi neurons did not rescue reductions in TDBTN, TDBL and soma size (Figs S2 and S3), suggesting that these key components of thiamine signaling are independently required for regulating correlated growth of dendrite arbors and neuronal somata.

### Pyrithiamine significantly suppresses dendrite morphogenesis and soma growth

**Figure 7:**
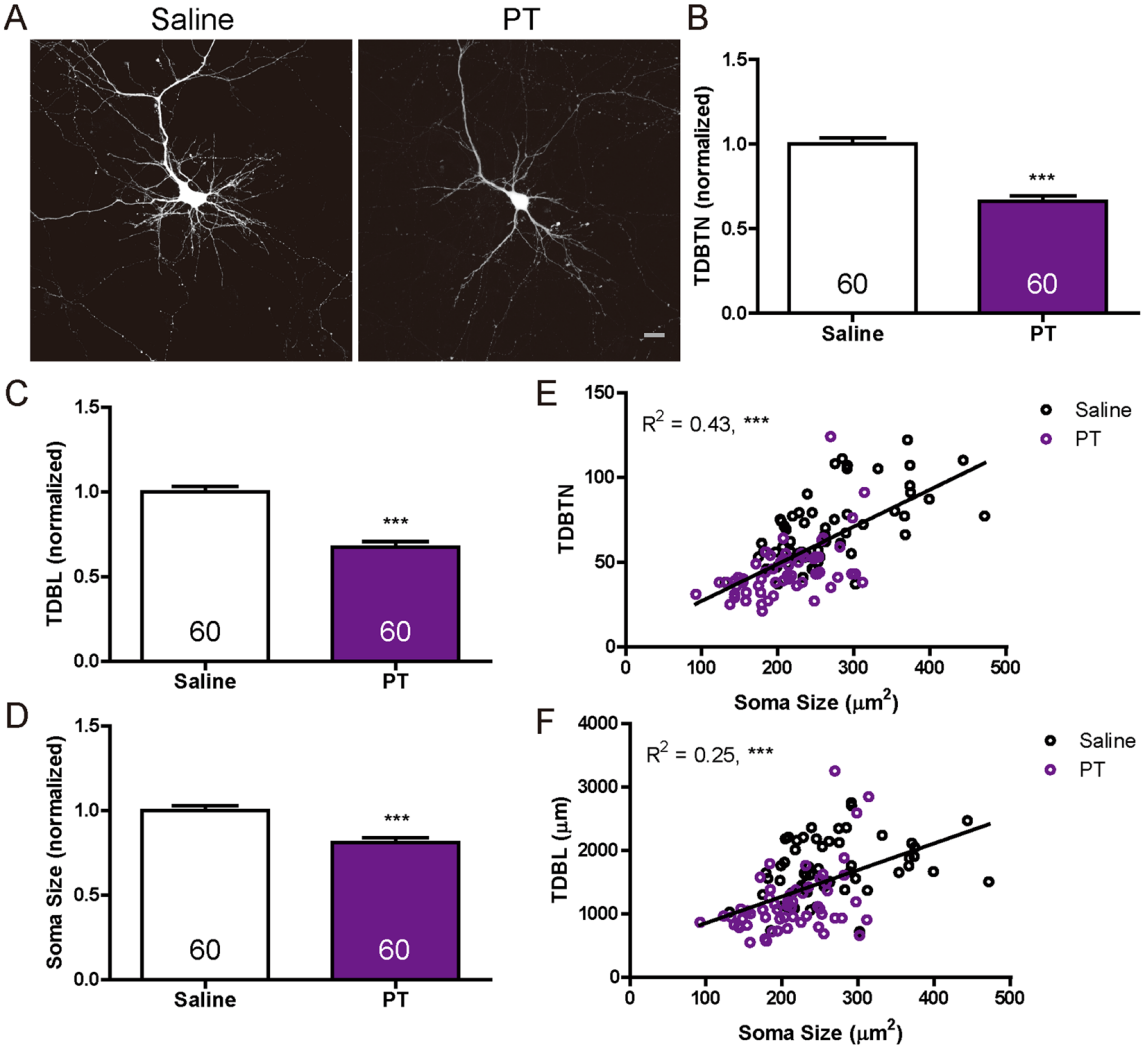
Pyrithiamine significantly reduced TDBTN, TDBL and soma size. (**A**) Representative images of hippocampal neurons treated with saline or Pyrithiamine (PT, 50 μM) for 48 hours (DIV7 – DIV9). Scale bar: 20 μm. (**B**) Quantitation of TDBTN: saline (1.00 ± 0.04), PT (0.66 ± 0.03, *P* < 0.001). (**C**) Quantitation of TDBL: saline (1.00 ± 0.03), PT (0.67 ± 0.03, *P* < 0.001). (**D**) Quantitation of soma size: saline (1.00 ± 0.03), PT (0.81 ± 0.03, *P* < 0.001). (**E**) A significant correlation between TDBTN and soma size (*n* = 120, R^2^ = 0.43, *P* < 0.001). (**F**) A significant correlation between TDBL and soma size (*n* = 120, R^2^ = 0.25, *P* < 0. 001).

By manipulating the intracellular levels of *Tpk1*, *Slc25a19* and *Slc19a3*, we identify a critical role of thiamine metabolism in regulating dendrite complexity and soma size. We further tested these effects using a complementary pharmacological approach, by treating neurons with pyrithiamine, a competitive substrate for Tpk1 that can be phosphorylated to form pyrithiamine diphosphate^18^ but cannot functionally substitute for TDP. Pyrithiamine treatment has been shown to induce symptoms similar to those of thiamine deficiency in animals^19^. Neurons were transfected with GFP on DIV 6, and treated with pyrithiamine (50 μM) from DIV 7 to DIV 9. As compared to the saline-treated group, pyrithiamine treatment significantly reduced TDBTN, TDBL and soma size (Fig. 7A–D). Furthermore, both TDBTN and TDBL significantly correlated with soma size (Fig. 7E,F). These results provide further support for the importance of thiamine metabolism to coordinately regulating dendrite arborization and soma size in developing neurons.

## Discussion

In this study, we investigated the role of thiamine metabolism in regulating early neuronal development at the cellular level. By manipulating the intracellular levels of Tpk1, Slc25a19 and Slc19a3, three proteins key to thiamine metabolism, as well as pharmacological blockade of this pathway, we identified a critical role of thiamine metabolism in regulating correlated growth of neuronal soma and dendrite arbors in hippocampal neurons. These results provide new insight into the contribution of thiamine metabolism to neuronal morphogenesis at the cellular level.

Dendrite arborization is a critical process to neuronal growth and development. Previous studies showed that it is regulated by a combination of extrinsic cues and intracellular molecules^20–23^, including neuronal activity, diffusible cues, cell contacts and transcription factors. Identified targets of these signals encompass the cytoskeleton, protein synthesis, membrane turnover, changes in genetic programing and so forth. Here we add to the list by showing that thiamine metabolism, which participates in cellular energy metabolism, also contributes to promoting dendrite outgrowth. While the importance of energy metabolism to cellular processes, including protein synthesis, transcriptional regulation and cytoskeletal dynamics, cannot be overstated, how they contribute to regulate neuronal morphogenesis is little known and could be the subject of future exploration.

While the close relationship between the somata and the dendrites is apparent, as the cell body needs to provide raw materials for the growth and arborization of dendrites, changes in cell size^24^ has rarely been studied in the context of dendrite development. The few studies that reported changes in both soma size and dendrite arborization^25–27^ did not examine whether these changes were correlated, and all focused on the PI3K-mTOR signaling pathway, which has established roles in regulating dendrite morphogenesis^20, 21, 23^ and cell size^24^. Thus, to our knowledge, we present the first demonstration of highly correlated changes in soma size and dendrite arborization. In terms of interactions with other pathways, we speculate likely interaction with the PI3K-mTOR signaling pathway, as mTORC1 integrates inputs from multiple cues that affect cell growth, including stress, energy status, oxygen, and amino acid levels^28^. Importantly, both pathways have the capability to regulate soma size and dendrite arborization. How precisely the regulation occurs and/or whether thiamine metabolism interacts with other known pathways regulating dendrite development and soma size remains to be determined in future studies.

An interesting question arising from our observations is which change occurs first, soma size or dendrite arbors? All three RNAi significantly reduced TDBTN and TDBL and soma size by DIV 12. The only effect observed at DIV 8 was a reduction in soma size in *Tpk1* RNAi neurons (Figs 1, 3 and 5). This result hints at changes in soma size preceding those in dendrites. Consistently, in absolute numbers, all three RNAi induced considerable reduction in soma size (value for DIV 16 lower than that of DIV 8), but only attenuated growth in TDBTN and TDBL (value for DIV 16 similar to that of DIV 8). Control neurons grew in all three parameters over the same time span. Together, these results are consistent with soma growth being a necessary prerequisite for dendrite arborization and outgrowth, and thiamine metabolism contributing to the process. Of the genes examined, the effects of *Tpk1* RNAi were most similar to those of pyrithiamine treatment, consistent with *Tpk1* being the rate limiting enzyme in thiamine metabolism in neurons.

The overexpression experiments, in addition to demonstrating specificity of the RNAi effects, also gave some insight into the effect of overexpressing components of thiamine metabolism. Overexpressing human *TPK1*, *SLC25A19* or *SLC19A3* significantly rescued the reduction in TDBL and TDBTN induced by their respective RNAi, and *TPK1* or *SLC19A3* overexpression alone also increased dendrite arborization (Figs 2, 4 and 6). The magnitude of the increases were generally smaller than the reductions induced by RNAi, suggesting that thiamine metabolism is important, but likely not rate limiting for dendrite growth. In terms of soma size, *TPK1* overexpression significantly rescued the effect of *Tpk1* RNAi, while *SLC19A3* overexpression alone induced a small increase in soma size. The difficulty to induce an increase in soma size through overexpression is consistent with it being a highly regulated process, and with thiamine metabolism being necessary but not sufficient for soma growth. The inability of *SLC25A19* or *SLC19A3* overexpression to rescue *Tpk1* RNAi suggest that these components of thiamine metabolism are independently and non-redundantly required for neuronal development.

Finally, in the context of developmental disorders associated with mutations in *SLC19A3*, *SLC25A19* and *TPK1*, including Leigh syndrome^5, 7, 12, 13^ and congenital microcephaly^14^, our results provide cellular evidence for how perturbations in thiamine metabolism can inhibit growth of dendritic arbors and reduce soma size, changes that contribute to smaller brain size and neuronal degeneration.

## Methods

### Animals

Animal procedures were approved by the Institutional Animal Care and Use Committee of the Institute of Neuroscience, Chinese Academy of Science, in accordance with the standards established by the US National Institutes of Health. All experiments involving animals were performed in accordance with these regulations.

### Neuronal culture

Primary hippocampal neuronal cultures were prepared from postnatal day 0 (P0) Sprague-Dawley rat pups as previously described^29–31^. Briefly, dissociated neurons were plated on matrigel- (BD Biosciences, Sparks, MD, USA) or PDL- (Sigma-Aldrich, St. Louis, MO, USA) coated glass coverslips (Assistent, Sondheim/Rhön, Germany) at 50,000 cells/cm^2^, in Neurobasal medium (Thermo Fisher Scientific, Waltham, MA, USA) containing B-27 (Thermo Fisher Scientific), 2 mM Glutamax-I (Thermo Fisher Scientific), and 2.5% FBS (GE Healthcare Life Sciences, Pittsburgh, PA, USA). On DIV 3, when the entire coverslip is covered by a monolayer of astrocytes, cells were treated with the mitotic inhibitor 5-fluoro-2′-deoxyuridine (Sigma-Aldrich).

### DNA constructs

RNAi constructs were designed as oligonucleotides (GenePharma, Shanghai, China) targeting both mouse and rat sequences of the gene of interest, and tested for knockdown efficiency by western blotting. The effective ones were then inserted into pSuper-GFP. Sequences used for morphological analyses include: (1) *Tpk1* RNAi-2 sequence: CCTGAAGTCAAAGAGTACTTT for mouse *Tpk1* (NM_013861) and rat *Tpk1* (NM_001134994) sequences; (2) *Slc25a19* RNAi-1 sequence: GGTATGAGCTCTTCTGTAATT for mouse *Slc25a19* (NM_026071) and rat *Slc25a19* (NM_001007674) sequences; (3) *Slc19a3* RNAi-1 sequence: TAACCTGAGCTTAGAACGTTA for mouse *Slc19a3* (NM_030556) and rat *Slc19a3* (NM_001108228) sequences. The other sequences tested by Western blotting, as shown in Supplementary Figure 1 are: (1) *Tpk1* RNAi-1: GAAGGGCTGTGATCTTATTTT; (2) *Slc25a19* RNAi-2: GGTACGAGCTCTTCTGTAATT; (3) *Slc19a3* RNAi-2: AGCCTACTTTGCCTACATATA.

Full-length mouse sequences (RNAi sensitive) were used to test RNAi efficiency, while the full-length human sequences (RNAi resistant) were used for rescue experiments. All sequences were PCR cloned from cDNA generated using mouse brain tissue or human HEK 293 cells and cloned into pCS2. For mouse *Tpk1* (NM_013861) and human *TPK1* (NM_022445), a Myc tag (8 repeats) was inserted C-terminal to the coding sequence to generate *Tpk1*-myc and *TPK1*-myc respectively. For mouse *Slc25a19* (NM_026071) and human *SLC25A19* (NM_001126121), an HA tag was added N-terminal to the coding sequence to generate HA-*Slc25a19* and HA*-SLC25A19*. Similarly, for mouse *Slc19a3* (NM_030556) and human *SLC19A3* (NM_025243), a N-terminal HA tag was added to generate HA-*Slc19a3* and HA-*SLC19A3*.

For measuring TDP level in cultured neurons, the *Tpk1* RNAi-2 sequence was inserted into the FUGW lentiviral vector to generate LV-*Tpk1* RNAi (Obio Technology, Shanghai, China), which expressed shRNA under the H1 promoter and EGFP under the Ubiquitin promoter.

### Transfection and pharmacology

At DIV 6, neurons were transfected using calcium phosphate^32^, a method that combines low transfection efficiency (about 1%) and a high rate of co-transfection^33^, well-suited for morphological analyses. 2–4 μg of DNA were used per coverslip, including 0.2–0.4 μg of morphology marker (GFP or tdTomato). The low ratio of morphology marker DNA as compared to the genes of interest ensure that all cells analyzed express the gene of interest^29^. Neurons were fixed between DIV 8 and 16, as indicated for specific experiments.

For pharmacological treatment, neurons were treated with 50 μM pyrithiamine (PT, Sigma-Aldrich) on DIV 7 and fixed on DIV 9. Pyrithiamine was dissolved in saline, and control neurons were treated with an equal volume of saline.

For western blots, N2A cells were cultured in high glucose Dulbecco’s modified Eagle medium (DMEM, Gibco, New York City, NY, USA), supplemented with F12 Nutrient Mixture (1:1, Gibco) and 10% FBS (GE Healthcare Life Sciences). Cell were transfected with Lipofectamine 2000 (Invitrogen, Carlsbad, CA, USA), using oligonucleotides (2–5 μg) and/or plasmid DNA (1–2 μg) and harvested 48 hours later.

To test TDP level following *Tpk1* RNAi, LV- *Tpk1* RNAi and control lentiviruses were added into culture medium on DIV 4 with a multiplicity of infection of 5 TU/cell. Cultures were harvested for high performance liquid chromatography on DIV 11.

### Immunocytochemistry

Hippocampal neurons were fixed in 4% paraformaldehyde (PFA, Sigma-Aldrich) in phosphate-buffered saline (PBS) for 20 min, permeabilized in 0.1% Triton-X100 in PBS for 5 min, and blocked in 3% bovine serum albumin (BSA, Calbiochem, San Diego, CA, USA) in PBS for 1 h at 37 °C. They were then incubated with primary antibodies in 3% BSA in PBS overnight at 4 °C and followed by secondary antibodies for 1 h at 37 °C, and mounted in Fluoromount-G (Electron Microscopy Sciences, Hatfield, PA, USA). Antibodies were as follows: anti-HA (rabbit, ab9110-100, 1:1000; Abcam Cambridge, MA, USA), anti-Myc (mouse, 9E10, 1:500; Developmental Studies Hybridoma Bank, Iowa City, IA, USA), goat anti-rabbit Alexa-Fluor 568 (A11036, 1:500; Thermo Fisher Scientific) and goat anti-mouse Alexa-Fluor 568 (A11031; 1:500; Thermo Fisher Scientific).

### Image acquisition and morphological analysis

Z-stack images were acquired on either a Zeiss LSM5 Pascal laser scanning confocal microscope (Carl Zeiss, Jena, Germany) or a Nikon A1 confocal microscope (Nikon, Tokyo, Japan), at 1 μm intervals, using a 40x oil immersion objective (N.A. = 1.3). Pyramidal neurons were discerned by their pyramidal morphology, as visualized by GFP or tdTomato. All protrusions longer than 3 μm were considered as dendritic branch tips and the projected Z-stacks were analyzed blinded to the experimental condition. Total dendritic branch tip number (TDBTN) and soma size were counted using ImageProPlus (Media-Cybernetics, Silver Spring, MD, USA), while total dendritic branch length (TDBL) was traced and measured using ImageJ software (NIH Image). All experiments were repeated with at least three independent culture preparations and normalized to neurons transfected with control constructs from the same culture preparation.

### Western blot

Western blot was performed according to standard protocols. The following antibodies were used: anti-TPK (rabbit, 10942-1-AP, 1:5000; Proteintech, Rosemont, IL, USA), anti-Myc (mouse, 9E 10, 1:2000; Developmental Studies Hybridoma Bank), anti-HA (mouse, M20003, 1:500; Abmart, Shanghai, China), anti-GAPDH (mouse, KC-5G4, 1:3000; Kangcheng, Shanghai, China), anti α-Tubulin (mouse, T6074, 1:100,000; Sigma-Aldrich), anti-mouse HRP (goat, AP308P, 1:10000; Millipore, Billerica, MA, USA), and anti- rabbit HRP (goat, AP307P, 1:10000; Millipore).

### High performance liquid chromatography

Primary hippocampal neurons infected with lentiviruses for 7 days were washed twice in PBS and scraped from 12-well plates carefully. Part of each sample was used for determining total protein concentration, and the rest immediately deproteinized with isometric 5.0% perchloric acid. After centrifugation at 13,000 g at 4 °C for 20 min, the supernatant were collected and the level of thiamine and its phosphate esters levels measured using high performance liquid chromatography (HPLC) as previously described^34^. Briefly, thiamine and its phosphate esters were derivatized into thiochromes using potassium ferricyanide. The derivatives were then separated by gradient elusion with a C18 reversed-phase analytical column (250 × 4.6 mm) and measured by HPLC fluoroscopy (Agilent 1100, Santa Clara, CA, USA) with an excitation wavelength of 375 nm and emission wavelength of 435 nm. TDP, TMP and thiamine levels were quantified using standard samples of TDP, TMP, and thiamine (Sigma-Aldrich). The HPLC operators were blind to sample information.

### Statistical analysis

Statistical analysis was carried out in GraphPad Prism 5 (GraphPad Software, La Jolla, CA) using unpaired two-tailed Student’s *t*-test (for sample pairs) or one-way ANOVA (for groups of three or more conditions) followed by Tukey’s multiple comparison test. For comparison between multiple time points, two-way ANOVA followed by Bonferroni’s multiple comparison test were used. Linear regressions were performed using GraphPad Prism 5. All data are represented as mean ± s.e.m., and “*n*” represents the number of neurons. All conditions statistically different from control are indicated: *P < 0.05; **P < 0.01; ***P < 0.001; n.s., not significant.

## Electronic supplementary material

Supplementary Figures
