# Supplementary material for: Thiamine metabolism is critical for regulating correlated growth of dendrite arbors and neuronal somata
Source: Sci Rep. 2017 Jul 13;7:5342. doi: 10.1038/s41598-017-05476-w (PMC5509691; doi:10.1038/s41598-017-05476-w)
Supplement: Supplementary file 1 — Supplementary Figures [file 41598_2017_5476_MOESM1_ESM.pdf]

Thiamine metabolism is critical for regulating correlated growth of dendrite arbors and neuronal somata

Huimin Liu[1, 2], Shaoming Sang[1], Yuan Lu[3], Zhongfeng Wang[1], Xiang Yu[3]*, Chunjiu Zhong[1]*

1. Institutes of Brain Science & Collaborative Innovation Center for Brain Science; Department of Neurology, Zhongshan Hospital; State Key Laboratory of Medical Neurobiology, Fudan University, Shanghai 200032, China.
2. School of Life Sciences, Fudan University, Shanghai 200438, China.
3. Institute of Neuroscience, State Key Laboratory of Neuroscience, CAS Center for Excellence in Brain Science and Intelligence Technology, Chinese Academy of Sciences, Shanghai 200031, China.

*Corresponding authors: Chunjiu Zhong (email: zhongcj@163.com)
Xiang Yu (email: yuxiang@ion.ac.cn)

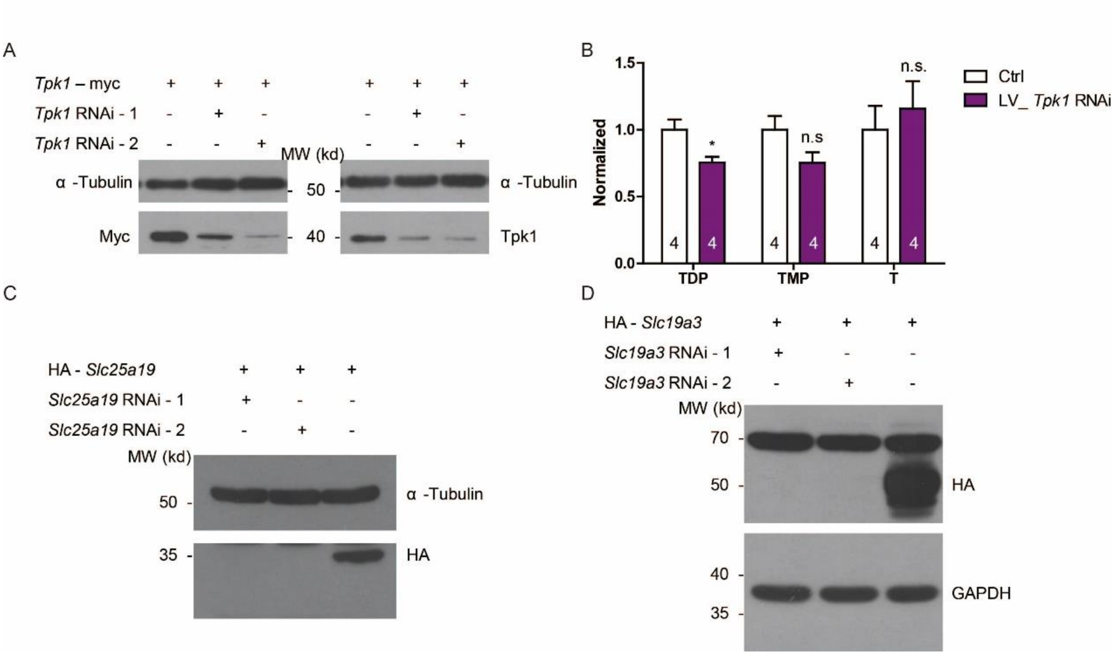

Supplementary Figure 1. Testing the knockdown effect of RNAi sequences. A. Western blot of Myc tag and Tpk1 level in N2A cells transfected with *Tpk1*-myc and/or *Tpk1* RNAi. B. TDP, TMP and T level in neurons infected by lentiviruses expressing control and *Tpk1* RNAi sequences from DIV4 to DIV11. TDP (0.75 ± 0.04, $P < 0.05$), TMP (0.75 ± 0.08, n.s.), T (1.16 ± 0.20, n.s.) in *Tpk1* RNAi neurons. N = 4 culture preparations, unpaired *t*-test. C. Western blot of HA tag in N2A cells transfected with HA-*Slc25a19* and/or *Slc25a19* RNAi. D. Western blot of HA tag in N2A cells transfected with HA-*Slc19a3* and/or *Slc19a3* RNAi. Cropped blots are presented here; full-length blots are presented in Supplementary Figure 4.

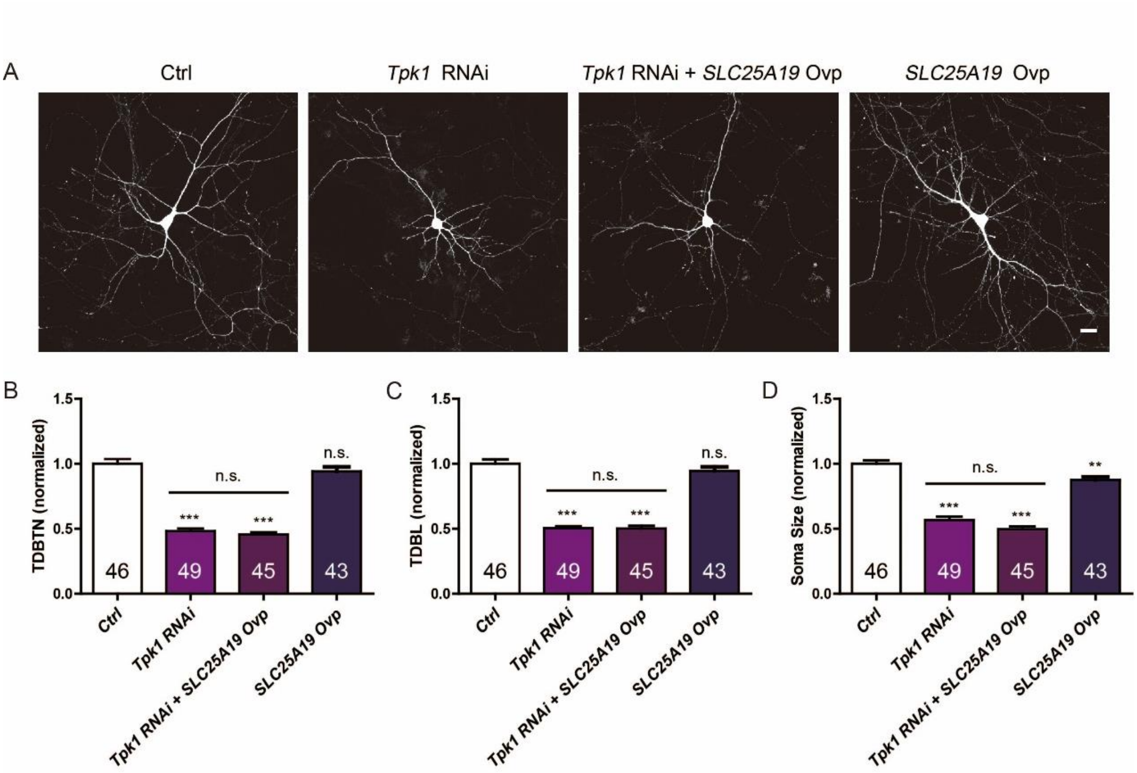

Supplementary Figure 2. Overexpression of human *SLC25A19* did not rescue the reduction of TDBTN, TDBL and soma size induced by *Tpk1* RNAi. A. Representative images of DIV10 neurons, conditions as indicated. B. Quantitation of TDBTN: Ctrl (1.00 ± 0.04), *Tpk1* RNAi (0.48 ± 0.02, $P < 0.001$ vs Ctrl), *Tpk1* RNAi + *SLC25A19* Ovp (0.46 ± 0.02, $P < 0.001$ vs Ctrl, n.s. vs *Tpk1* RNAi), *SLC25A19* Ovp (0.94 ± 0.03, n.s. vs Ctrl). C. Quantitation of TDBL: Ctrl (1.00 ± 0.03), *Tpk1* RNAi (0.51 ± 0.01, $P < 0.001$ vs Ctrl), *Tpk1* RNAi + *SLC25A19* Ovp (0.50 ± 0.02 $P < 0.001$ vs Ctrl, n.s. vs *Tpk1* RNAi), *SLC25A19* Ovp (0.95 ± 0.03, n.s. vs Ctrl). D. Quantitation of soma size: Ctrl (1.00 ± 0.03), *Tpk1* RNAi (0.57 ± 0.02, $P < 0.001$ vs Ctrl), *Tpk1* RNAi + *SLC25A19* Ovp (0.50 ± 0.02, $P < 0.001$ vs Ctrl, n.s. vs *Tpk1* RNAi), *SLC25A19* Ovp (0.88 ± 0.02, $P < 0.01$ vs Ctrl). One-way ANOVA followed by Tukey's post-test. Scale bar: 20 μm.

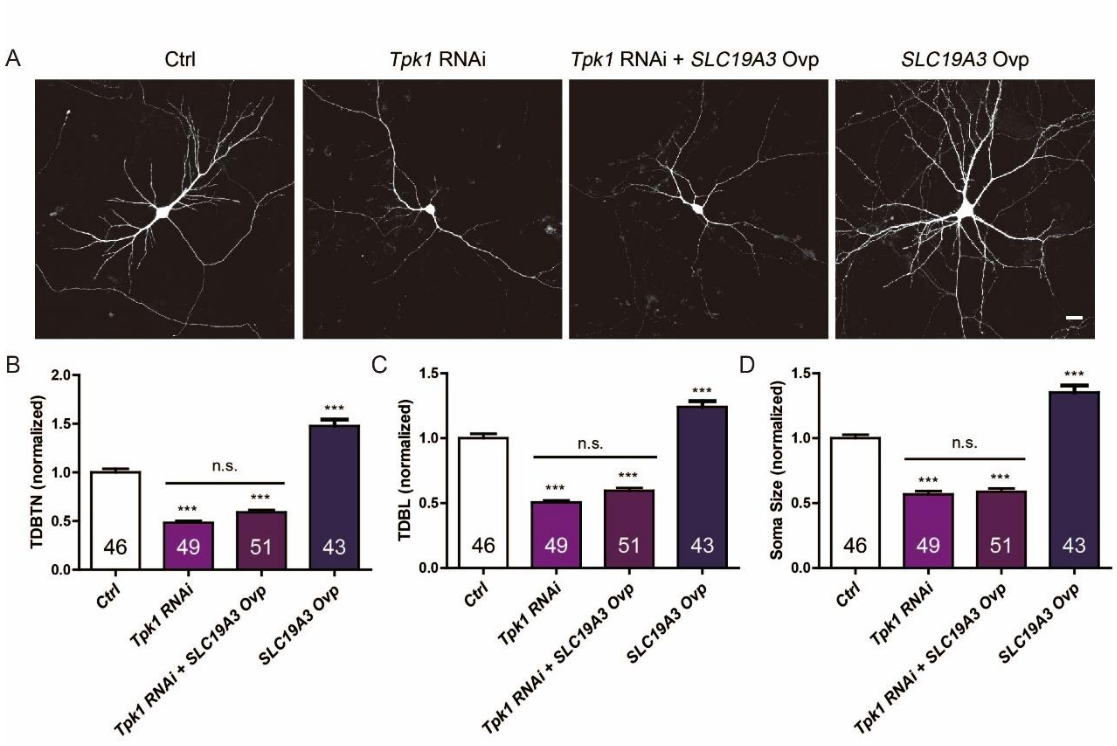

Supplementary Figure 3. Overexpression of human *SLC19A3* did not rescue the reduction of TDBTN, TDBL and soma size induced by *Tpk1* RNAi. A. Representative images of DIV10 neurons, conditions as indicated. B. Quantitation of TDBTN: Ctrl (1.00 ± 0.04), *Tpk1* RNAi (0.48 ± 0.02, $P < 0.001$ vs Ctrl), *Tpk1* RNAi + *SLC19A3* Ovp (0.59 ± 0.02, $P < 0.001$ vs Ctrl, n.s. vs *Tpk1* RNAi), *SLC19A3* Ovp (1.48 ± 0.07, $P < 0.001$ vs Ctrl). C. Quantitation of TDBL: Ctrl (1.00 ± 0.03), *Tpk1* RNAi (0.51 ± 0.01, $P < 0.001$ vs Ctrl), *Tpk1* RNAi + *SLC19A3* Ovp (0.60 ± 0.02 $P < 0.001$ vs Ctrl, n.s. vs *Tpk1* RNAi), *SLC19A3* Ovp (1.24 ± 0.04, $P < 0.001$ vs Ctrl). D. Quantitation of soma size: Ctrl (1.00 ± 0.03), *Tpk1* RNAi (0.57 ± 0.02, $P < 0.001$ vs Ctrl), *Tpk1* RNAi + *SLC19A3* Ovp (0.59 ± 0.02, $P < 0.001$ vs Ctrl, n.s. vs *Tpk1* RNAi), *SLC19A3* Ovp (1.35 ± 0.05, $P < 0.001$ vs Ctrl). Data for control and *Tpk1* RNAi neurons are the same as that shown in Supplementary Figure. 2. One-way ANOVA followed by Tukey's post-test. Scale bar: 20 μm.

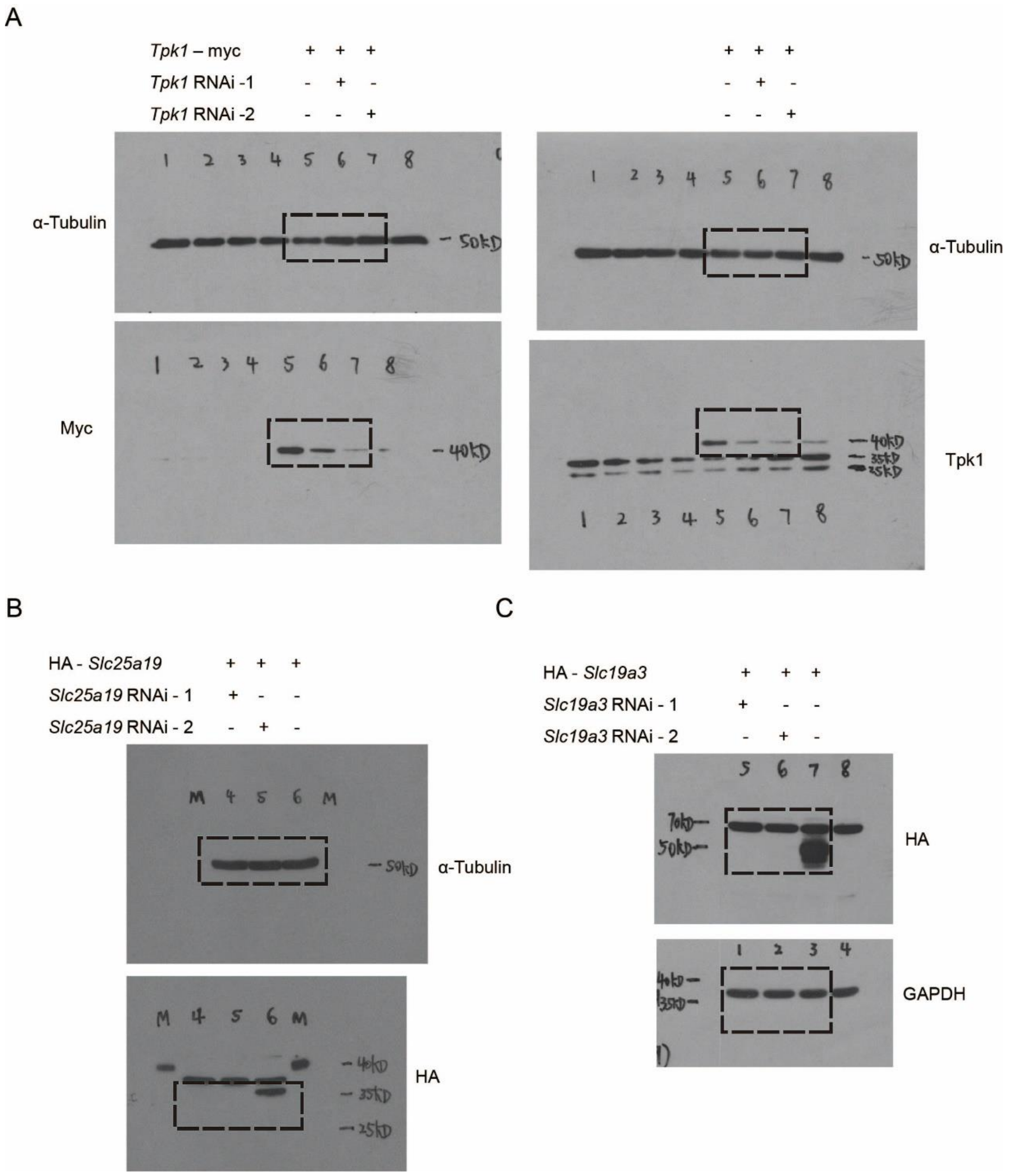

Supplementary Figure 4. Full-length images of immunoblots presented in supplementary Figure 1. Full-length images of cropped immunoblots presented in Fig. S1A (A), Fig. S1C (B) and Fig. S1D (C) are shown.
